# Mapping and quantifying the spatial and temporal composition of waste piles in informal settlements of urban Malawi

**DOI:** 10.1007/s11356-026-37534-0

**Published:** 2026-03-07

**Authors:** Taonga Mwapasa, Tony Robertson, Dyson Kazembe, Andrew Mnkhwamba, Patrick Ken Kalonde, Nicholas Feasey, Richard S. Quilliam, Tracy Morse, Kondwani Chidziwisano

**Affiliations:** 1 Centre for Water, Sanitation, Health, and Appropriate Technology Development (WASHTED), Malawi University of Business and Applied Sciences (MUBAS), Blantyre, Malawi; 2 Biological and Environmental Sciences, Faculty of Natural Sciences, University of Stirling, Stirling, UK; 3 Malawi Liverpool Wellcome Programme, Kamuzu University of Health Sciences, Blantyre, Malawi; 4 Department of Vector Biology, Liverpool School of Tropical Medicine, Liverpool, UK; 5 Department of Clinical Sciences, Liverpool School of Tropical Medicine, Liverpool, UK; 6 School of Medicine, University of St Andrews, St Andrews, UK; 7 Department of Civil and Environmental Engineering, University of Strathclyde, Glasgow, UK; 8 Department of Public and Environmental Health Sciences, Malawi University of Business and Applied Sciences, Blantyre, Malawi

**Keywords:** Environmental pollution, Informal settlements, Plastic pollution, Single-use plastic, Solid waste, Urban waste piles, Waste composition

## Abstract

Solid waste pollution is a multifaceted global challenge affecting the environment, economy, and human health, yet our understanding of environmental solid waste dynamics, such as waste type, weight(W), and volume(V), which inform waste management initiatives and policy frameworks remains limited in Sub-Saharan Africa. In this longitudinal study, we mapped informal waste disposal sites using transect-walks and drones in urban Malawi. Monthly characterisation of selected individual waste piles was undertaken for 12 months. Sampling (200L/waste pile) included both surface and compacted sections. (60 cm depth), which were characterised by weight(kg/m^2^), volume(m^3^), and individual counts of plastic items(m^2^). Three-way ANOVA and a linear mixed-effect model were used to predict percentage recovery for the interactions between month, waste pile depth, and waste type as fixed factors. Fourteen out of 56 waste piles received detailed temporal characterisation. Waste piles were located along rivers(50%), streams(29%), and open-dumping spaces(21%). Soil (W:60%, V:19%), organic material (W:22%, 33%), and plastics (W:8%, V:24%) were the highest components by weight, and volume, making plastics the highest non-organic category of waste. Seven plastic polymer types were identified, with Low-Density Polyethylene being the most common (83% plastic items/m^2^). Seasonality, waste pile depth, and waste type (F(171, 4284) = 11.44, p < .001, η^2^ = .31) significantly affected percentage recovery of waste items, indicating their effect on waste pile dynamics. Sustainable waste management initiatives need to account for seasonal changes, material characteristics, and waste pile stratification to effectively improve waste management systems and the public health implications of waste piles.

**Supplementary Information:**

The online version contains supplementary material available at 10.1007/s11356-026-37534-0.

## Introduction

Across the globe, over two billion tonnes of municipal solid waste is generated every year and this is estimated to increase to 3.7 billion tonnes by 2050 if no urgent action is taken (UNEP & ISWA 2024b). The sub-Saharan Africa (SSA) region alone generated over 200 million tonnes of solid waste in 2020, the majority of which was uncontrolled (UNEP & ISWA 2024b). SSA currently ranks the lowest in solid waste collection, with only 36% of its generated waste collected. This is even less in Malawi, with only 25% of the total waste generated in urban settings collected for safe disposal, primarily from planned settlements in higher income areas (Kasinja & Tilley, 2018). The absence of formal waste management systems and weak enforcement mechanisms in Malawi has led to the prevalence of open dumping of waste and the creation of informal dump sites/waste piles within the wider community; these sites are often situated along roadsides or water bodies, thereby causing direct pollution to the environment (Kasinja & Tilley, 2018; Mpanang’ombe et al. 2021; Turpie et al. 2019).

Similar to other countries in SSA who have recorded organic waste as the highest waste component (Adedara et al. 2023; Kassahun et al. 2025; UNEP & ISWA 2024b), Malawi’s waste generation rate is estimated to be 0.42–0.55 kg/per person/day, mostly dominated by organic waste and plastics(Gondwe et al. 2019). This is compounded by a deficit in material recovery strategies such as composting and recycling, which results in the high accumulation of waste in the environment (Turpie et al. 2019). Nevertheless, Low- and Middle-Income Countries (LMICs) such as Malawi can leverage material recovery from waste as this offers both environmental and economic benefits. Currently Malawi, like many other countries, lacks up-to-date data on waste volume, composition and quality which is vital in planning for material recovery and other waste management initiatives (UNEP & ISWA 2024b). Furthermore, Malawi also lacks proper waste separation at source for domestic solid waste, which can in turn affect the quality of recovered waste and material recovery efficiency. For example, the lack of separation of organic waste, such as food waste, leads to the contamination of other waste such as plastics and cardboard, potentially making their recycling less viable (Kaza et al. 2018).

High-income countries produce and consume significantly more plastic per capita than LMICs, but are also better equipped to manage plastic waste due to well-developed infrastructure and resources (Halog & Anieke 2021; UNEP 2024a, b). With 10% of solid waste estimated to be composed of plastics (20 million tonnes in SSA per year) in 2020, this weight is only set to increase in coming years (Adedara et al. 2023; Kaza et al. 2018). This is of particular concern in unplanned (informal) urban settlements in SSA, where rapid population growth and urbanisation result in increased plastic waste generation (Babayemi et al. 2019; Heidbreder et al. 2019; Kibria et al. 2023; Lebreton & Andrady 2019).

Across SSA, illegal dumping has been attributed to poor urban planning, non-collection of waste and weak enforcement of regulations, resulting in widespread pollution and risks to both human and animal health (Kasinja & Tilley, 2018a; Mphasa et al. 2025; White et al. 2023). It is therefore crucial for urban settings to effectively manage solid waste, including plastic waste, to minimise its negative impacts. Studies have been undertaken on illegal solid waste dumping sites (waste piles) to understand their geolocations, and community perceptions towards illegal dumping with the primary aim of informing potential interventions in waste management. (Jakeni et al. 2024; Muheirwe et al. 2023; Ngalo & Thondhlana 2023). However, there is limited information on the composition of waste in these illegal dumpsites, with previous studies focused on household level sampling, often over short periods of time, not taking account of the informal waste picking which could affect the waste disposed of in these primary sites. (Dladla et al. 2021; Jakeni et al. 2024; Kibria et al. 2023; Ngalo & Thondhlana 2023). Seasonal variations such as rainfall patterns, temperature shifts, and changes in consumption behavior, can also significantly influence both the quantity and composition of waste (Chen et al. 2020; Kamran et al. 2015; Zia et al. 2017) especially in dumpsites which serve different populations and are exposed to environmental and climatic changes.

The first step is identifying the scale of the problem to inform context appropriate solutions; however, there is limited quantitative data on solid waste composition and plastic waste dynamics in SSA cities. Such data could be used to inform and promote waste management initiatives such as waste reduction, recycling, and resource recovery through a circular economy approach. These data are also critical for informing the design of effective solid waste management interventions including challenges and opportunities for transportation, collection, recycling, and composting efforts (Thyberg & Tonjes 2015; Ziraba et al. 2016). We speculated that, given the high level of poverty in high density residential areas of Blantyre (Malawi), where waste collection services are insufficient, there were likely to be a high number of informal waste piles, which were seasonal in nature, and were likely to be composed of waste materials which had no obvious route for reuse or recycling. Therefore, our aim was to conduct a longitudinal study to characterise and quantify solid waste composition in informal dumping sites in urban Blantyre. Specifically, we aimed to: (1) spatially map areas of informal waste dumping; and (2) quantify the temporal dynamics of the composition of urban waste piles, with a specific focus on plastic waste.

## Materials and methods

### Study setting

**Fig. 1:**
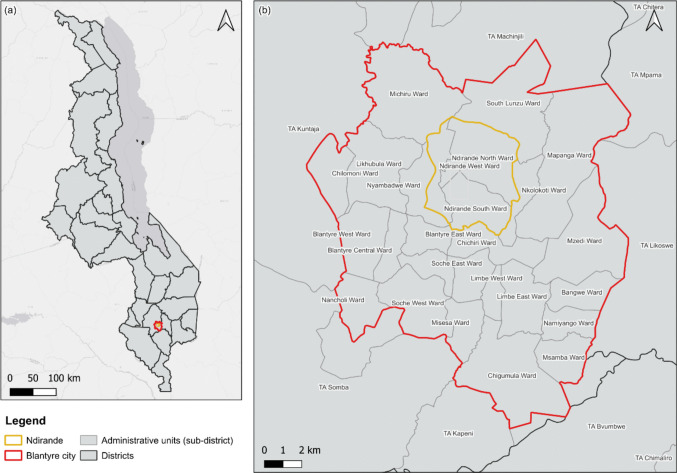
Map of the study area. **a** shows the location of Blantyre City (in red) relative to other districts in Malawi; (**b**) highlights the location of Ndirande (in gold) within Blantyre City (in red). Source: Authors own generated figure

The study was conducted in Ndirande township, an urban informal settlement in Blantyre, Malawi. Ndirande has an area of 14.33 km^2^ and a population of approximately 118,000 people, which is estimated to be 15% of the total population of Blantyre (National Statistical Office 2019). The township is characterized by a high population density with most of the settlement being unplanned (Fig. 1) (Cocker et al. 2022). The unplanned nature of the settlement limits formal waste collection services, which leads to open dumping of domestic solid waste into household environments, streams, and surrounding rivers (Turpie et al. 2019).

### Data collection

**Fig. 2:**
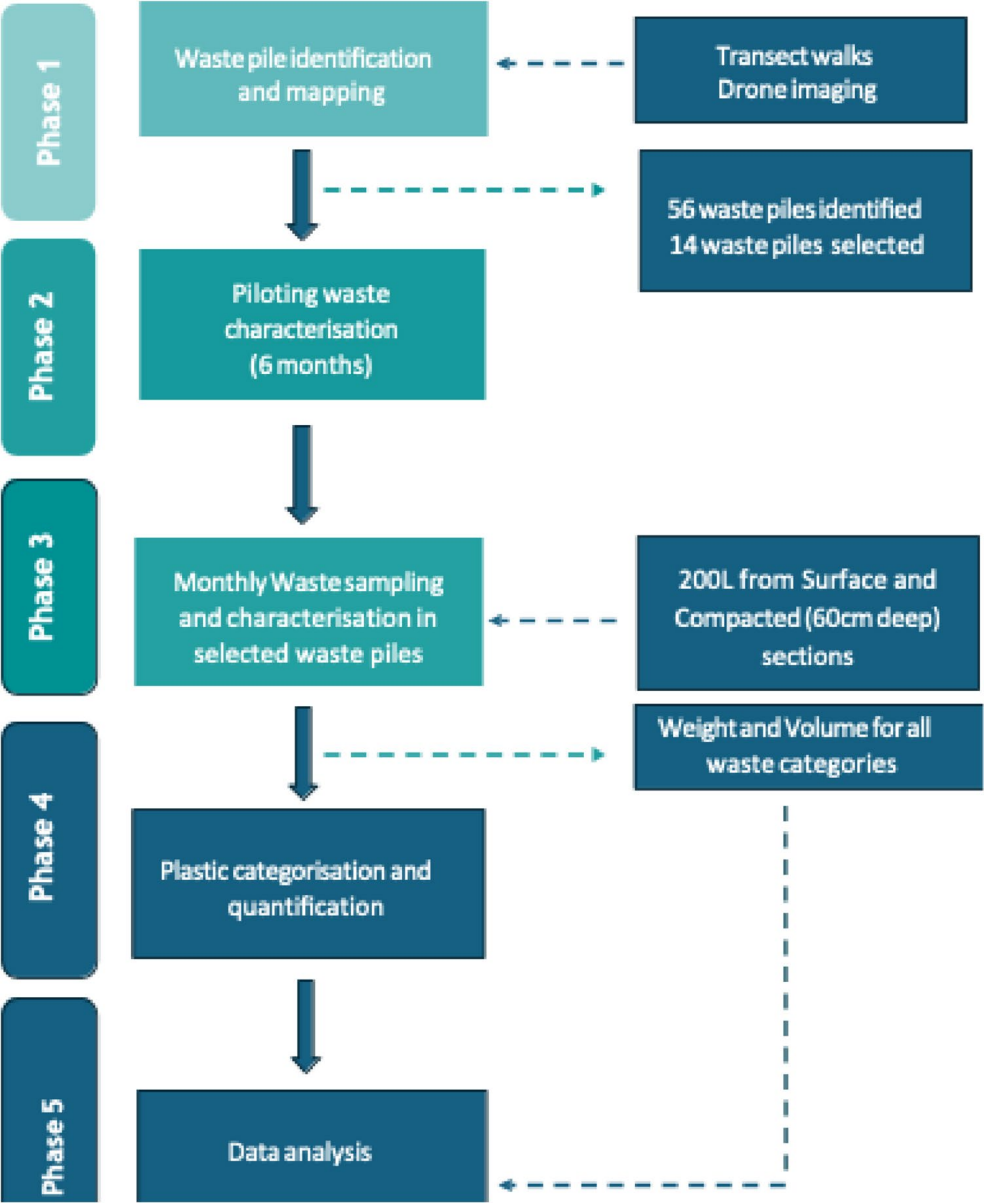
Graphical workflow of the study methodology

Data collection was conducted in four phases (Fig. 2): (1) waste pile identification and mapping, which then informed, (2) waste characterisation piloting, before embarking on, (3) longitudinal waste sampling and characterisation process, and (4) plastic categorisation and quantification. Data collection for phases 3 and 4 were concurrent. Mapping and identification of waste piles were conducted from November and December 2021, and the methodology of characterising waste categories designed and tested from December 2021 to June 2022. Waste characterisation data were then collected monthly from July 2022 to June 2023, and the mean monthly rainfall, recorded at Ndirande automated rainfall station, was compiled by the Department of Climate Change and Meteorological services in Malawi.

Data collection was conducted by a five-member team which consisted of three researchers and two community members (identified by the community leader). Prior to the commencement of data collection, community leaders, Health Surveillance Assistants (HSAs), and the Village Development Committee members (VDC) were separately engaged through individual meetings. This was done to ensure wide community sensitization on the study process and facilitate greater acceptance of the study among community members. Features and interviews on local radio programmes were also used to sensitize the wider population.

#### Phase 1: waste pile identification and mapping

Waste pile identification and mapping took place from November to December 2021 across the whole of Ndirande. Initial transect walks targeted rivers, streams, and open spaces. The research team walked through the study site to identify areas of open waste dumping, and drone imagery was used to validate waste pile sites from these walks. Drone images were captured by a DJI Mavic 2 Enterprise equipped with a 12-megapixel camera (aperture range f/2.8–3. 8), from an altitude of 60 m (Kalonde et al. 2025). During the walks, the team collected data electronically on a standard checklist form in KoBoCollect to record details such as: accessibility of the waste pile for study equipment (such as wheelbarrows, sorting tables, gazebo, buckets, shovels etc.), location of waste pile (river, open space, or streams), proximity to households, and geo-location of the pile. Each waste pile was assigned a unique identification number.

#### Phase 2: waste characterisation piloting

**Fig. 3:**
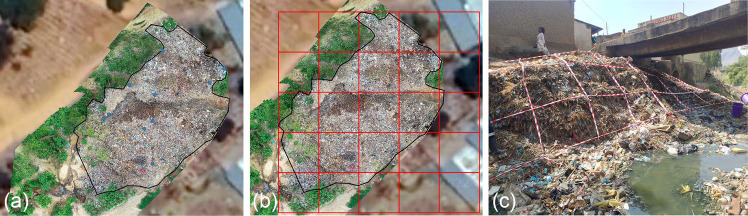
Randomized sample collection process. **a**) Randomised sample points (blue dots) on a waste pile **b**) Randomised sample points and grid formation using GIS **c**) On-the-ground manual grid formation and sampling at each waste pile. Source: Authors own photograph

Sampling and characterisation of waste were based on principles from the Integrated Solid Waste Management Plan Manual by the United Nations Environmental Programme (UNEP/IETC 2009). The toolkit for measuring waste was adapted from the Chartered Institution of Wastes Management (CIWM) and WasteAid UK tools (Lenkiewicz & Webster 2017). The method was piloted and refined in fifteen waste piles during January 2021 to June 2022 to ensure that it was appropriate for the local settings. During piloting, 200L of waste was sampled at each waste pile for six months of the methodology development to ensure that the sample weight was within the 50–100 kg range as recommended (Lenkiewicz & Webster 2017) and volume samples were opted over weight samples to avoid moisture content bias during seasonal comparison and to reduce the weight bias between surface and compacted waste pile sections. A depth of 60 cm was also determined to represent the compacted section because waste was consistently observed to be compacted at this level, and it offered a safe excavation limit given the slanted structure of some piles (e.g., Fig. 3); excavating deeper could have compromised pile stability. This depth was also practical and reachable with the available equipment (e.g., shovels). Similar studies have used depths of around 0.5 m (50 cm) among other depths to assess waste pile/dumpsite age and stratification (Gyabaah et al. 2023), supporting the suitability of 60 cm as a reasonable depth for examining compacted sections of waste piles. To ensure reliability of the data, a mass balance check was performed for each waste sample. Total pre-sorting weight was compared with the sum of all sorted fractions to assess mass loss during waste characterization and all samples included in the analysis had mass losses within the acceptable threshold (≤ 3%). Prior to waste characterization, all nearby households/individuals were briefed on the planned waste characterization to ensure that the community was not suspicious or disruptive to the activity.

#### Phase 3: waste sampling and characterisation process

The waste characterisation and plastic quantification process took place once every month for twelve months at fourteen selected waste piles, ensuring sampling for each location was approximately four weeks apart. Equipment for waste characterization was set up near the waste pile, including a gazebo, tables, scale, buckets, and sample collection tools such as rakes, wheelbarrow, and shovels. The size of the waste pile (length and width), location (river, stream, or open space), proximity to households, and GPS coordinates (with accuracy of < 10 m), were recorded on an electronic form using KoBo Collect. To ensure representative sampling of the waste pile, and effective waste sorting, the Chartered Institute of Waste Management (CIWM) toolkit’s principles were followed. Initially, Geographic Information System (GIS) and drone imagery were utilized to map the full size of each waste pile (Fig. 3a), creating 1.5 m by 1.5 m grids (Fig. 3b) and random sampling points assigned within these grids. However, limitations such as internet connectivity when using the GPS Essential Application (https://www.gpsessentials.com/) to locate sampling points led to the adoption of a manual grid formation method, i.e., tape was used to manually create 1.5m^2^ grids on the waste pile, and eight random points for waste sample collection were selected (Fig. 3c). Two composite samples per waste pile (one for “surface waste”, one for “subsurface/compacted waste”) were obtained per month, each comprising of 100L, making a total sample size of 200L per waste pile. Wastes were collected across the eight randomly selected sampling grid points at each waste pile.,. Surface waste was loosely positioned on top of the pile representing recently deposited waste. Compacted waste had undergone compression and potentially decomposition and was collected at a depth of 60 cm from the waste pile surface. Surface waste from each of the eight identified sampling points were combined until 100 L volume was reached.. Subsequently a composite sample of compacted waste was collected from the same points by digging to the required depth. Twelve composite samples of “surface waste” and twelve composite samples of “subsurface/compacted waste” were collected per waste pile in twelve months.

Both the surface and compacted waste samples were weighed and sorted separately into ten waste categories: organics, plastics, metal, glass, paper and cardboard, sanitary items, fabric/textile, foil (coated plastic and aluminum packaging), soil and fine particles (i.e., < 1 cm), and other. Waste was sorted immediately after recording weight after sample collection, and surface waste was first sorted and characterised prior to collecting of the compacted waste sample for sorting. Waste from each category was placed in 20 L buckets, and the weight and volume recorded.

#### Phase 4: plastic categorisation and quantification

**Fig. 4:**
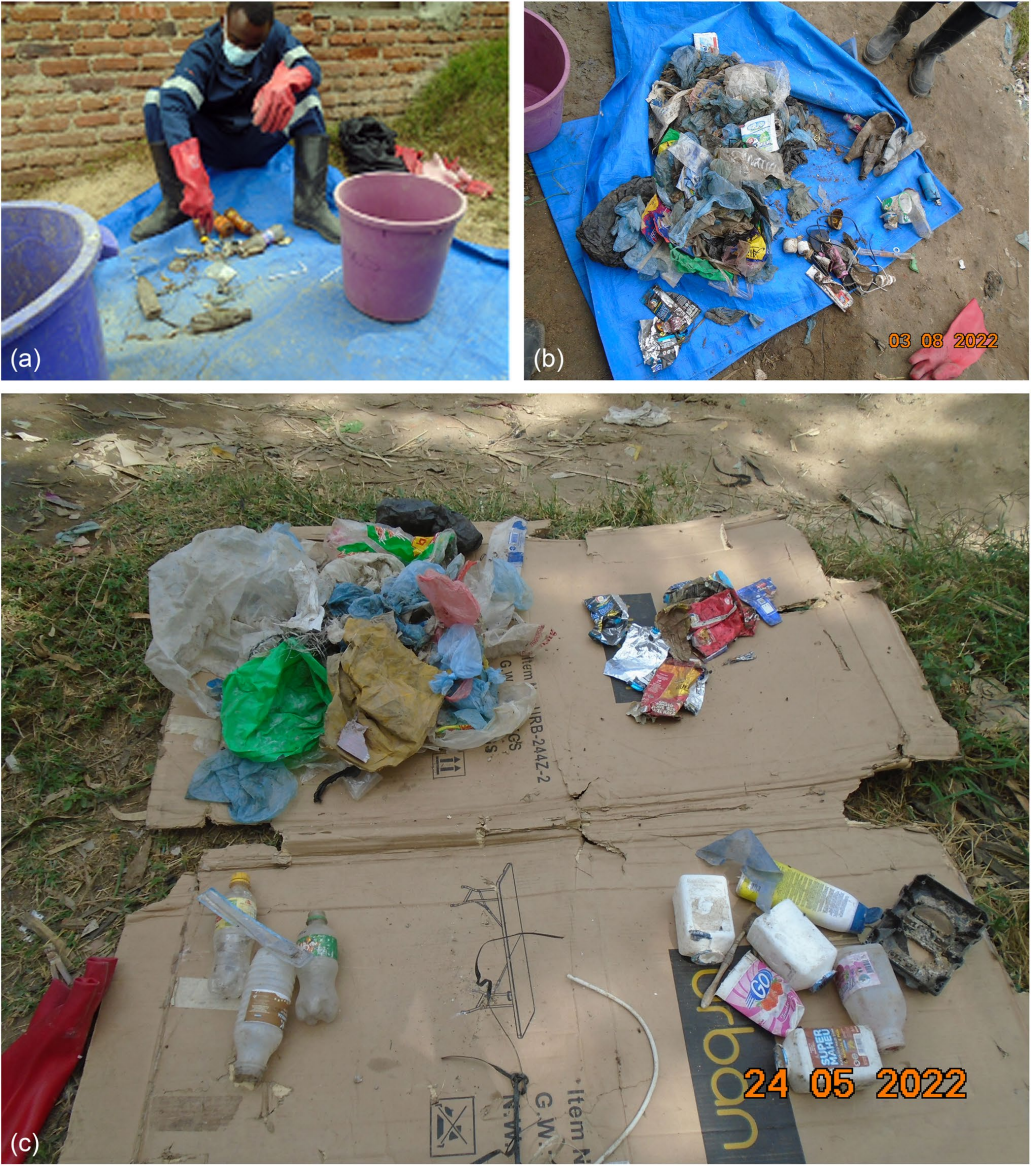
Manual plastic waste quantification by polymer type (**a**) research team member counting plastic polymers (**b**) Mixed plastic polymers during manual counting (**c**) separated plastic polymers during counting. Source: Authors own photograph

After waste sorting, the plastics from each waste pile were further categorized according to their different polymer types: Low-Density Polyethylene (LDPE), High-Density Polyethylene (HDPE), Polyethylene Terephthalate (PET), Polyvinyl Chloride (PVC), Polypropylene (PP), Polystyrene (PS) and other polymers (items made of a mixture of plastic and other components e.g. foil), through manual counting of each available plastic object (Fig. 4) to give insights into the frequency of plastic use regardless of mass which may be affected by polymer type and material size. Plastics from the surface and compacted sections were recorded separately at each waste pile. For polymer identification, the study team took time to explore examples of the different plastic polymers during the piloting period, and which identified a relatively small group of plastics being consistently found in waste piles making visual identification realistic for the longitudinal stage. To support this, a laminated copy of the different plastic polymers with common examples were available for reference in case of uncertainty. during Phase 4. Where uncertainty could not be resolved samples were placed in the category other/unknown polymers.

### Data analysis

#### Geospatial mapping and descriptive characteristics of waste piles

Waste pile coordinates collected using a checklist during transect walks were downloaded from KoBoCollect in comma-separated values (CSV) file format in Microsoft Excel and converted to Keyhole Markup Language (KML) files. Geolocations of waste piles were then plotted on the Ndirande map using QGIS 3.24.1 ‘Tisler’ (QGIS, 2022) application to show distribution of waste piles within the study site. Quantitative data related to the waste pile location, proximity to households and accessibility (collected using electronic checklists), was cleaned and coded using IBM SPSS version 28.0 (Armonk, NY: IMB Corp) statistical package, and descriptive univariate analyses conducted to generate frequencies and percentages. To allow comparison of waste quantities across sites, the weight of each waste category was normalized per unit area by dividing the category weight (kg) or plastic counts by the total sampled area (18 m^2^, total area for 8 (1.5 m × 1.5 m) grids), expressed as kg/m^2^ or item/m^2^ for plastics.

#### Statistical analysis

We conducted a three-way mixed factor ANOVA in R (v. 4.3.1) to determine how Month (12 levels), waste type (10 levels) and waste pile depth (2 levels) influenced waste quantity/recovery. Missing data were omitted from the model and the analysis focused on 4,284 data points. Assumptions of normality and homogeneity were assessed using Shapiro wilk test (*p* > 0.05) and Levene’s test (*p* > 0.05). A Gaussian linear mixed model with identity link was fitted and effect size were reported as Partial Eta squared to highlight the level of effect of each factor on waste recovery. To account for repeated measurements within piles, a linear mixed-effects model was fitted with random intercepts for waste pile.. The dependent variable was the percentage of recovery of waste volume and the fixed effects included month, waste type, and waste pile section/depth (surface and compacted), as well as their interaction, to assess the temporal, compositional, and vertical differences in waste recovery. In order to capture non-linear seasonal patterns found in the data, month was treated as an ordered factor and modelled using polynomial contrasts (linear through fifth order). Waste type and waste pile section were included as categorical fixed effects. The sampling unit was added as a random intercept to account for non-independence within piles since waste was repeatedly sampled from the same waste piles over several months. This approach allowed us to quantify both main effects and interaction effects while accounting for the hierarchical sampling structure of the data. Factors explaining differences in plastic proportion (Sect. "Plastics as a proportion of all waste") were explored using linear mixed models in SPSS (v. 28.0). The dependent variable used was the percentage of waste that was made up of plastics by both weight and volume (for both surface and compact samples). The main independent variables tested were month, which was treated as a repeat measure, and waste location (alongside a river, stream, or an open site away from a water body). These factors were included as fixed effects, including an interaction term. We allowed for random intercepts for the waste piles. Additional model adjustments were made, including mean monthly rainfall and mean monthly temperature as covariates in additional iterations of the baseline model. Sensitivity analysis swapped total monthly rainfall for mean monthly rainfall. One outlier was removed from the analysis and, due to missing data for the open waste pile sites and the relatively small sample size, we focused only on those waste piles (*n* = 11 waste piles) with 12-months of data.

### Ethical approval and safety

Ethical approval for the study was obtained from College of Medicine Research Committee (COMREC) under approval #P.07/20/3089, clinical trial number; not applicable. Permission was also sought from the Blantyre City Council who are responsible for waste management in Blantyre City. Community clearance for conducting the research was provided by community leaders through the Group Village Headmen (GVH). Safety training for all field workers was conducted prior to data collection, which included, the use of Personal Protective Equipment (PPE), safe waste handling, and hand hygiene; safety equipment was carried at all times.

## Results and discussion

### Waste pile mapping and description

**Fig. 5:**
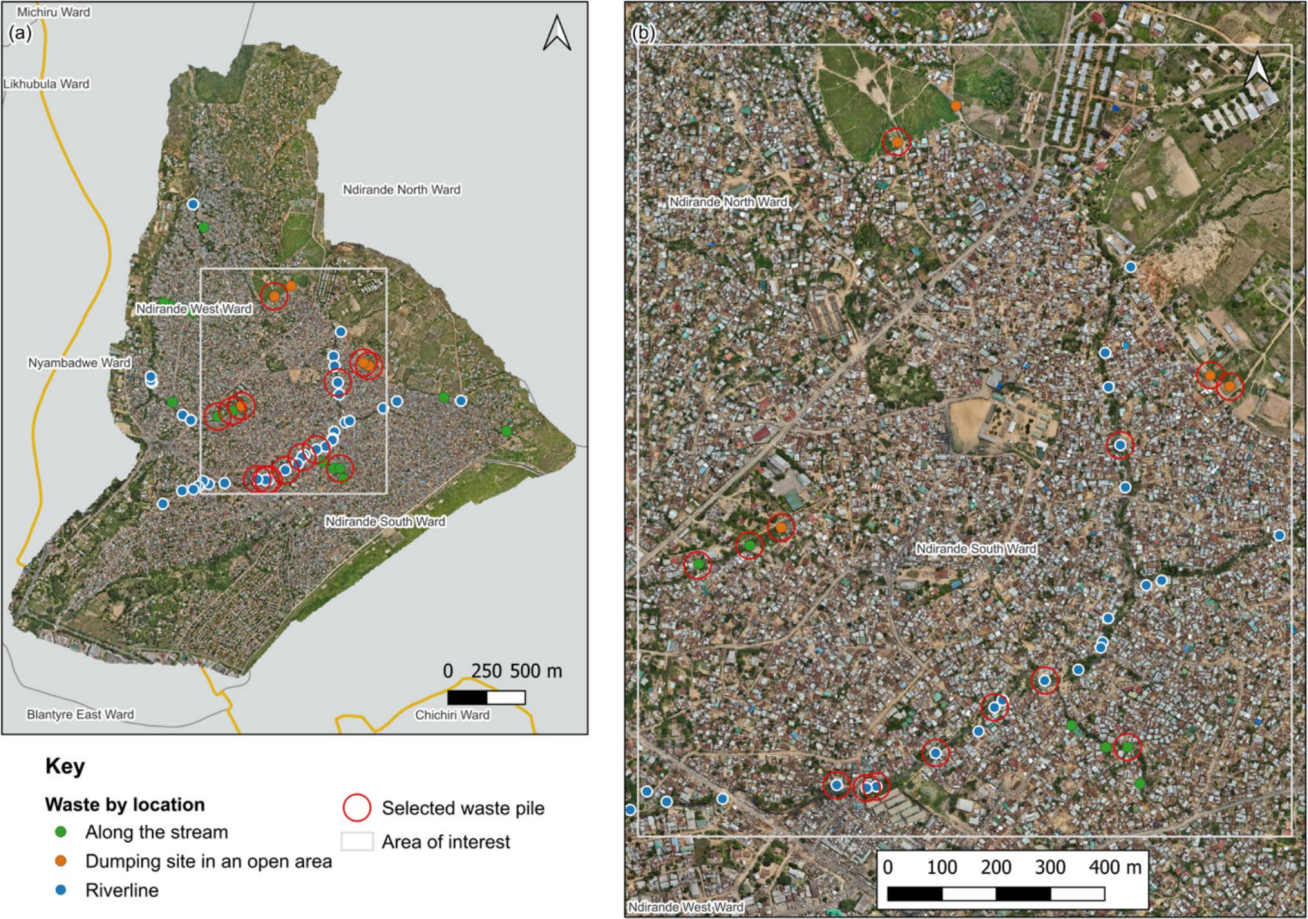
Map of Ndirande showing (**a**) locations of waste piles (*n* = 56) identified during transect walks; (**b**) waste piles selected for waste characterization (*n* = 14) based on inclusion criteria. Source: Authors own photograph

All identified waste piles were located within the Ndirande North, and West wards, which are the most densely populated areas, housing over three quarters of the population. A total of 56 waste piles were mapped (Fig. 5a), which were located along the Nasolo river-line (*n* = 36), streams (*n* = 15) and open dumping spaces (*n* = 5). Out of these, 14 waste piles were purposively selected (Fig. 5b) for detailed analysis based on their ease of access by the research team, safety during operations, and presence of enough space for sorting the waste within a 10 m radius of the waste pile. The selected waste piles were located in open dumping spaces (*n* = 3), alongside the river (*n* = 7), or by streams (*n* = 4) (Fig. 5). All selected waste piles were situated within 10 m of surrounding households. Waste pile size varied across months (Supplementary material S1) with the highest recorded waste pile area of 825m^2^ and the lowest area was less than 20m^2^.

Such close proximity of households to waste piles, puts people at potential risk of infectious disease transmission, with evidence from previous studies demonstrating that people living within ~ 50 m of dumping sites are at higher risk of suffering from waste related infectious diseases (Munyai & Nunu 2020; Okpara et al. 2021). Importantly, enteric bacterial pathogens such as *Salmonella typhi, Shigella spp.,* and *Vibrio Cholerae*, and multi-drug resistant Extended Spectrum β-lactamase (ESBL) producing *E.coli* and *Klebsiella Pneumoniae* were concurrently isolated from these same identified waste piles (Mphasa et al. 2025), highlighting the health risk that these communities are exposed to in the presence of these waste piles, which calls for urgent waste management initiatives. Moreover, most of the identified waste piles were along rivers and streams, similar to other studies which mapped waste piles in informal settlements of Blantyre (Kalonde et al. 2025; Mvula et al. 2025). Such open dumping of waste along rivers and open land can significantly increase the spread of infections by contaminating both water sources and soil (Ali et al. 2025; Farooq et al. 2023; Gautam et al. 2025; Njewa et al. 2025). In Blantyre for example, the rise in typhoid and cholera cases has been linked to the use of contaminated surface water such as river water, which could be associated with open dumping (Gauld et al. 2020, 2022; Stout et al. 2025). Similar patterns have been reported in other countries across southern Africa and parts of Asia, where unmanaged waste disposal contributes to surface-water contamination (Farooq et al. 2023; Gautam et al. 2025; Njewa et al. 2025). These findings highlight the substantial public-health risks posed by open waste dumping and underscore the need for improved waste management systems as the presence waste piles in Malawi, like other SSA countries, has been attributed to high urbanisation, limited waste management resource, lack of proper waste management systems, and recycling infrastructure among other things (Adedara et al. 2023; Okpara et al. 2021; Turpie et al. 2019).

### Waste composition

All selected waste piles contained examples from each of the ten waste categories (Table 1) from both the surface and the compacted sections. A total of 10,193 kg (29,800 L) of waste was sorted during this study; the minimum weight of waste sorted per waste pile was 21 kg (200L) and the maximum was 118 kg (200L), all results are reported on wet waste basis, with varying total weight of waste across the waste piles (Supplementary material S2). The waste stream in this study was dominated by soil and fine particles (≤ 1 cm), which accounted for 60.3% by mass but only 19.0% by volume, followed by the organic fraction constituting 22.2% by mass and 32.9% by volume, and plastics which contributed 8.1% by mass but 23.6% by volume. Glass (0.7% by mass; 0.5% by volume) and metal (0.4% by mass; 0.7% by volume) were the least dominant categories (Table 1). Overall, waste categories recorded fluctuating weight across months (Supplementary material S3) and occupied varying waste volumes (Table 1, Supplementary material S4). Waste densities varied across the surface and compacted sections of the waste piles with organics and plastics having lower waste densities and soil and fine particles the highest.

**Table 1:** Composition of waste by mass and volume within waste piles

Category (with examples)	Composition by mass (kg/m^2^)	Composition by volume (m^3^)	Waste density (kg/m^3^)	
			**Surface**	**Compacted**
**Biowaste/Organics** *Food waste, garden waste, agricultural waste*	22.2%	32.9%	9.6	17.8
**Paper & cardboard** *Newspaper, cardboard, magazines, office paper*	0.8%	4.2%	4.3	10.8
**Glass** *Clear or coloured bottles, composite glass*	0.7%	0.5%	19.4	25.7
**Metal** *Tin/steel containers, aluminium containers, other ferrous metal, other non-ferrous metal*	0.4%	0.7%	10.8	14.5
**Plastics** *(PET): Water and soda bottles, food containers* *(PVC): Cables, plumbing pipes* *(LDPE): Grocery bags, packaging* *(HDPE): Lotion bottles, packaging* *(PP): Bottle caps, medicine bottles* *(PS): Disposable cups, cutlery, packaging foam,* *Other polymers*	8.1%	23.6%	4.8	8.2
**Textiles** *Clothing, rags, blankets*	4.1%	6.8%	8.5	13.5
**Sanitary Waste** *Diapers, sanitary pads, cotton*	1.5%	6.0%	5.2	4.8
**Foil** *Crisps and snacks packaging*	0.3%	1.8%	2.6	4.7
**Other** *Mesh, rubber*	1.8%	4.4%	6.9	10.1
**Soil and fine particles** *Soil and particles of* ≤ *1 cm*	60.3%	19.0%	76.1	55.5

These findings highlight the variation in how different waste categories contribute to the waste stream: organics and plastics occupy the highest volume, whereas soil and fine particles contribute the most weight (mass), reflecting their substantially higher densities. The high composition of soil and fine particles by weight likely comes from the unpaved surfaces that these waste piles are located in and the degradation processes within the waste pile, contrary to other urban waste characterisation studies which collect samples directly from households resulting in less or no soil and fine particles category within their waste streams (Aslani & Taghipour 2018; Roman et al. 2025; Semilu Rosesar & Andari Kristanto 2020; Win et al. 2024). However, the large composition of organic waste followed by plastics aligns with similar trends in other low-income urban communities where food waste dominates due to subsistence lifestyles and limited food preservation technologies, and plastics due to increased use of packaged and single-use materials (Aslani & Taghipour 2018; Roman et al. 2025; Semilu Rosesar & Andari Kristanto 2020; Win et al. 2024). The low presence of glass, and metals could be attributed to informal recycling by waste pickers, who typically target valuable items such as glass bottles and metal containers before waste reaches disposal points (Gutberlet & Uddin 2017; and Kasinja & Tilley 2018). Together, these patterns highlight how material density, environmental context, and informal segregation and recovery practices shape the composition and physical characteristics of waste piles in informal settlements. While most studies report of waste weight only, which is crucial for waste management planning such as waste collection equipment, waste volume data are also critical for tracking progress in waste management assessments for environmental impacts and helps in identification of material consumption pattern which can be used to inform waste recovery (Kihila et al. 2021; Zhang et al. 2020).

**Fig. 6:**
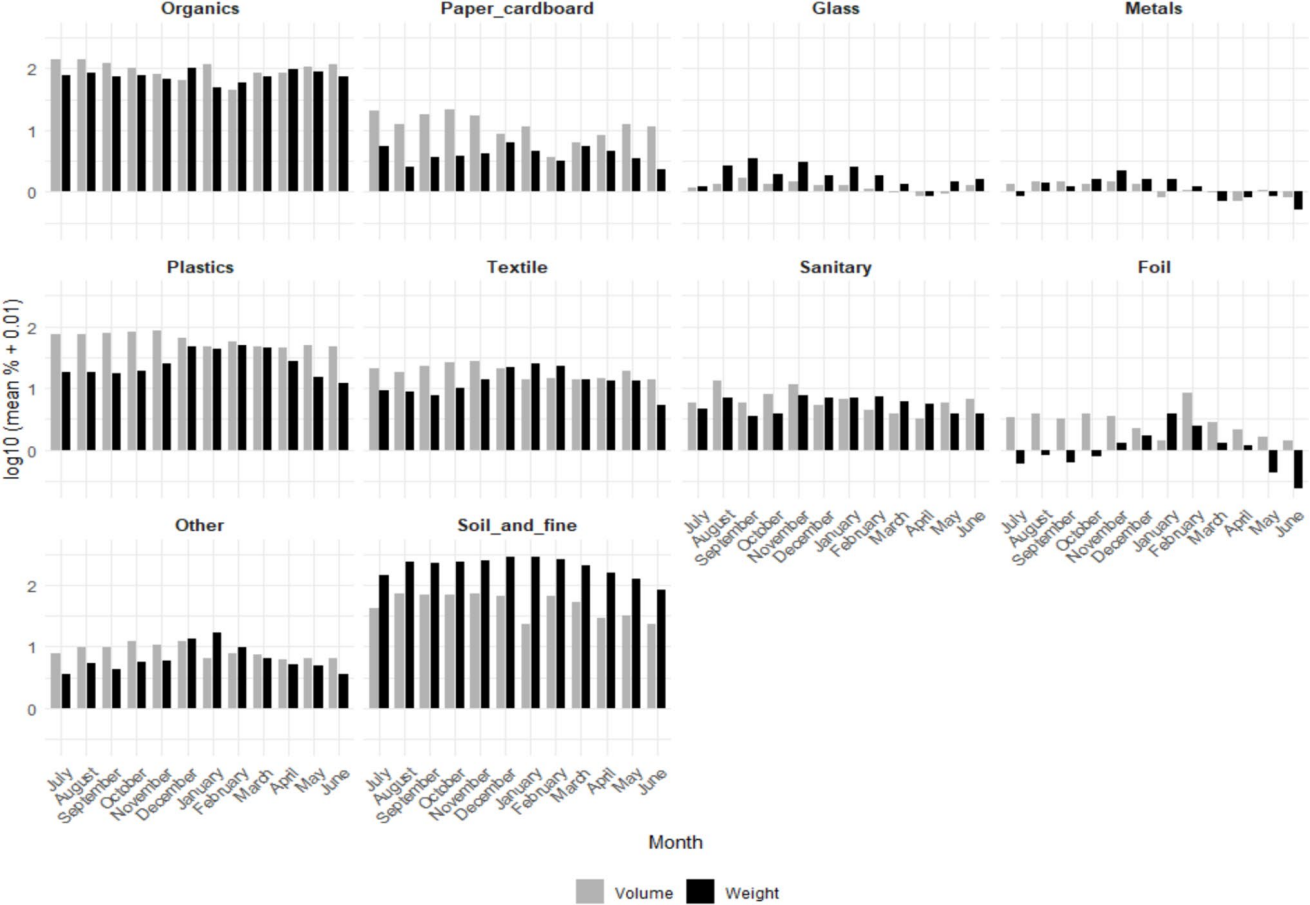
Monthly mean waste composition (log_10_ mean % + 0.01), across 14 waste piles in one year

**Fig. 7:**
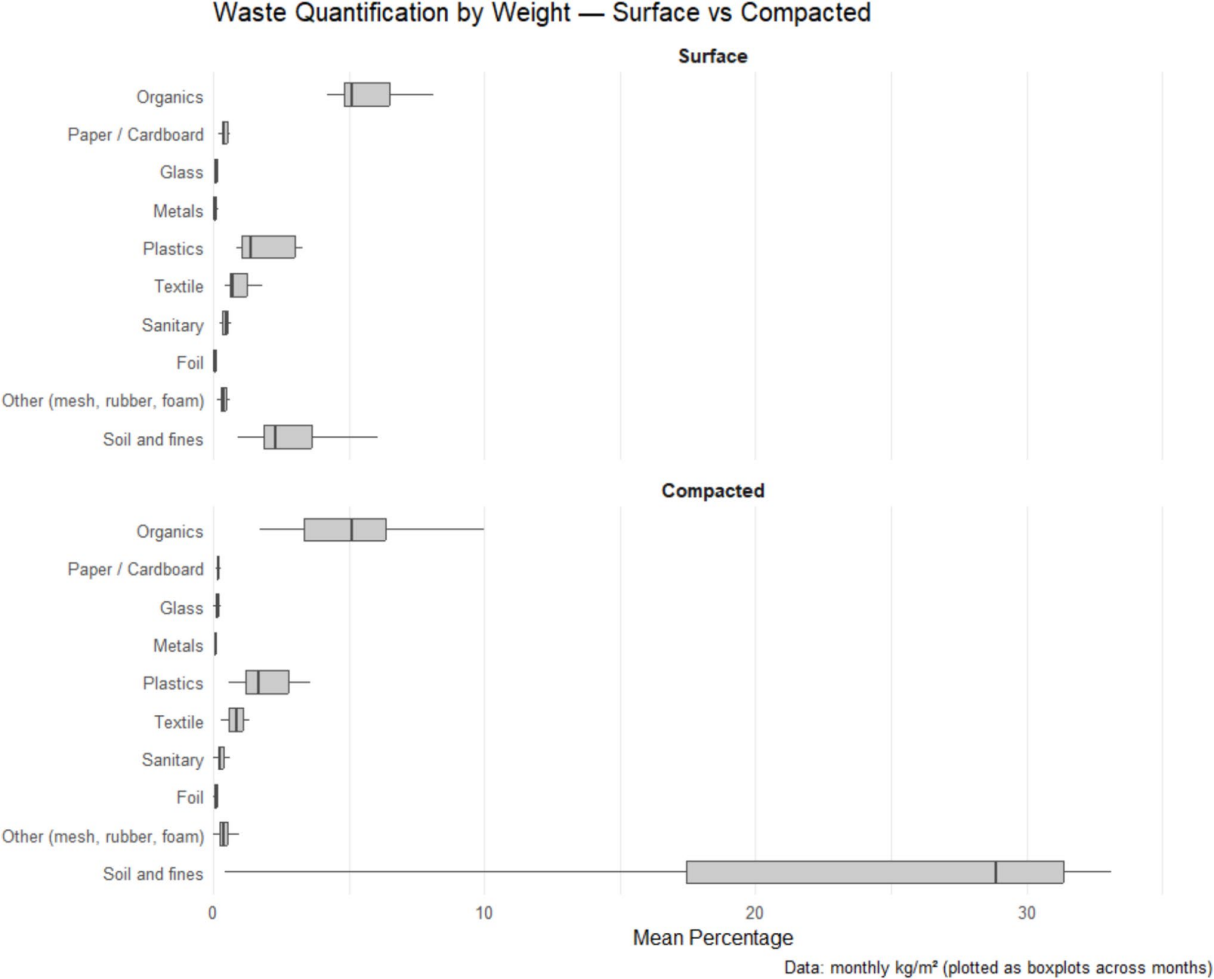
Weight of waste in surface and compacted waste pile sections

The mean weight and volume show that foil, glass, and metals were relatively low contributors of the waste piles over time (Fig. 6). Overall, organic material had the highest consistency in both weight and volume across months, while soil and fine particles had higher weight than volume. On the other hand, plastics, and paper had higher volumes than weight across the year. In contrast, glass, metals, sanitary waste, and foil display very low monthly values, often ≤ 0 on the log scale, indicating that these waste categories occur only in small quantities each month. There were some variations in the weight of waste materials between the surface and compacted sections of the waste pile, with notable variations between organic waste, and soil and fine particles (< 1 cm) and plastics (Fig. 7). The weight of organic waste was higher in surface waste compared to the compacted section, reflecting the frequent dumping of organic waste (such as food waste), whilst the weight of soil and fine particles, and plastics was higher in the compacted section reflecting the waste compression over time, making them dense. Less variations in weight were observed for categories with lower abundance such as, metals, glass, sanitary waste, and foil.

Overall, the monthly trends demonstrate that the waste stream is dominated by organics throughout the year, while other waste types fluctuate depending on seasonal consumption patterns, household behaviors, and collection practices. The use of a log scale is therefore important, as it allows meaningful comparison across months and categories that differ by a large difference in scale.

### Predictors of waste volume quantities in waste piles

**Fig. 8:**
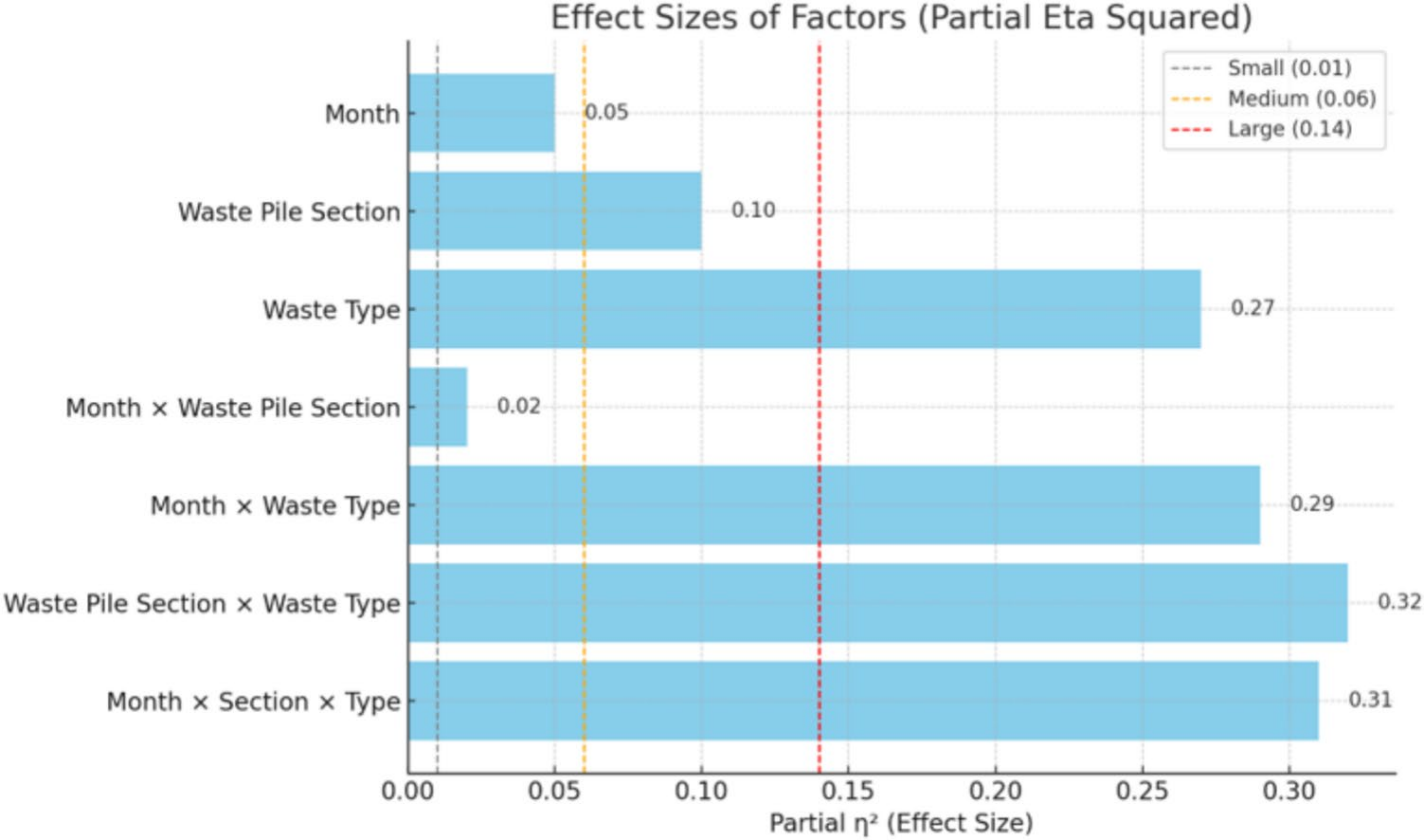
Effect Sizes of Factors used for waste volume percent (%) recover

The three‐way ANOVA highlighted a high three-way interaction between month × waste pile section × waste type (F (171, 4284) = 11.44, *p* < 0.001, η^2^ = 0.31) indicating the effect of the different factors on waste volume recovery, evident in the Partial Eta Squared results (Lakens 2013) (Table 2, Fig. 8).

**Table 2:** Three-way ANOVA for percent recovery of waste

**Source**	**df**	**SS**	**MS**	***F***	***p***	**Partial** *η*^**2**^
Month	11	3 950	359	19.30	< 0.001	0.05
Waste pile section	2	9 037	4 519	242.89	< 0.001	0.10
Waste type	9	28 795	3 199	171.98	< 0.001	0.27
Month × Waste pile section	19	1 789	94	5.06	< 0.001	0.02
Month × Waste type	99	33 127	335	17.99	< 0.001	0.29
Waste pile section × Waste type	18	37 598	2 089	112.28	< 0.001	0.32
Month × Waste pile section × Waste type	171	36 383	213	11.44	< 0.001	0.31
**Residual**	4284	79 696	19

These findings were confirmed by a linear mixed-effects model with random intercepts for each sampling location (Table 3). For example, the fixed estimate for organic matter was β = 3.19 (SE = 0.45), t (4600) = 7.13,* p* < 0.001, indicating that organic matter yields were on average 3.19% higher than plastics. Waste pile section had no significant effect (p = 0.603) on predicting percent recovery of waste. Seasonal trends in percent recovery based on “month” was non-linear, shown by the significant effects of both the linear trends (*p* = 0.012) and the fifth order polynomial term (*p* = 0.002) suggesting a complex (fluctuating) pattern with peaks and troughs in waste recovery rates across the year, rather than a simple monotonic trend.

**Table 3:** Selected fixed effects estimates from linear mixed-effects model predicting percent recovery

Predictor	Estimate	SE	*t*	*p*
**(Intercept)**	1.88	0.37	5.07	< 0.001
**Linear trend (Month.L)**	–2.78	1.10	–2.53	0.012
**5th‐order trend (Month**^**5**^**)**	3.52	1.11	3.18	0.002
**Waste pile section**				
Compacted	–0.43	0.82	–0.52	0.603
**Waste Type**				
Organics	3.19	0.45	7.13	< 0.001
Metals	–1.67	0.45	–3.72	< 0.001
Glass	–1.68	0.45	–3.74	< 0.001
Paper/Cardboard	–1.48	0.45	–3.29	0.001
Textile	–0.71	0.45	–1.58	0.114
Foil	–1.55	0.45	–3.46	< 0.001
Soil and fines	1.68	0.45	3.75	< 0.001
Other (mesh, rubber, foam)	–0.76	0.45	–1.70	0.09

Reference levels: Month = January (intercept), Waste pile section = Surface, Waste type = Plastics

Although variation in waste composition by mass and volume is expected across the different waste categories, this multi-factor ANOVA analysis helps to quantify the degree of these differences. Findings in this study demonstrate that single factors such as waste-pile section and month on their own might not fully influence percentage recovery of waste. However, a combination or two or more factors such as waste type, month and waste pile section together largely affect the percentage recovery of waste in waste piles. This interaction suggests that determinants of waste volume and weight in waste piles are more complex than simple descriptive comparisons suggest. Similar to our findings, previous studies have reported seasonal or monthly variation in waste quantities, without examining month (season) with other factors (Agrawal et al. 2013; Agwe et al. 2025; Singhal et al. 2022). And while fewer studies quantify vertical heterogeneity of waste at different depths (0.5m, 1.0m and 1.5m) in landfills or dumpsites (Gyabaah et al. 2023), our three-way interaction (month × pile section × waste type) provides a novel statistical confirmation of how seasonal (monthly), spatial and material factors jointly shape volumetric and compositional dynamics of waste, a complexity that single factor analyses may not fully capture.

### Plastic quantification

**Fig. 9:**
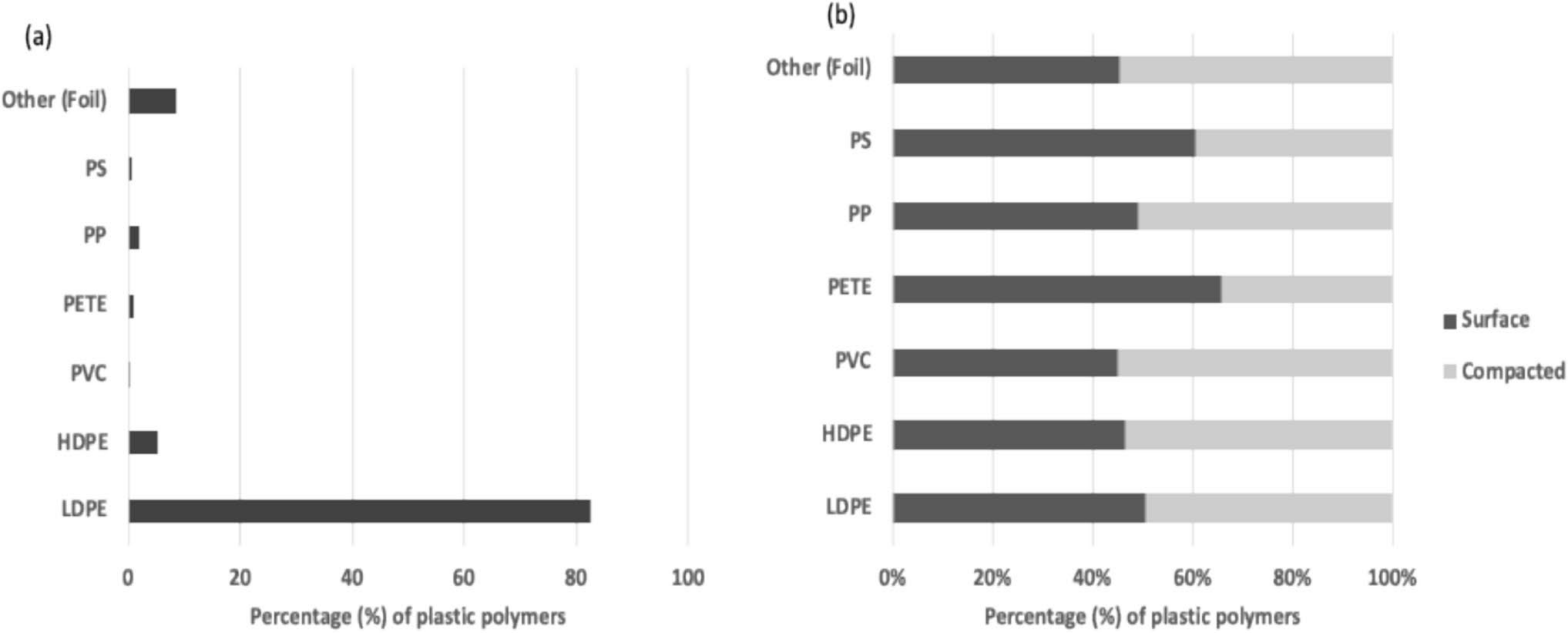
**a** Summary of cumulative plastic waste frequency (from counts of individual items) over a 12-month period from 14 waste piles (**b**) distribution of the total plastic frequency (in 8a) across the surface and compacted (sub surface) waste

Seven plastic polymer types were identified: LDPE, HDPE, PVC, PP, PS, PET, and other polymers (e.g., plastic-foil composites). In terms of individual items, the most frequent plastic in waste piles was LDPE (83%), indicating extensive use and disposal of lightweight plastics such as plastic bags and packaging, while PS (0.6%), PVC (0.2%) and PET (0.9%) were rarely found, likely due to either limited consumption or effective scavenging and recovery, particularly for PET, which is frequently collected for reuse and recycling before reaching disposal sites. (Fig. 9a). There was varying distribution of the different polymer types from the surface and the compacted areas of the waste pile which made up the total polymer quantities in Fig. 9a (Fig. 9b), and over the whole 12-month period and across waste piles (Supplementary Material S5-S9).

The difference in distribution of polymer types across the surface and compacted waste pile sections suggests temporal and recovery-related patterns. These findings show that LDPE were recorded in closely equal quantities in both the surface and compacted sections, reflecting its persistent and continuous disposal from plastic bags, packaging and films that enter the waste stream unchanged over time. In contrast, HDPE, PP, PVC, and other polymers, were more abundant at the surface, suggesting more recent disposal, changes in consumption patterns, and higher likelihood of material being recovered by waste pickers over time (Kasinja & Tilley, 2018). On the other hand, PET and PS were more common in compacted waste, likely representing older waste layers where these polymers were previously disposed at its end of life, as current PET items are increasingly recovered at the surface due to their high reuse and recycling value. These patterns highlight the potential differences in consumer use trends, recovery practices, and the persistence of specific polymer types within the waste system.

While there is less detailed research on the quantification of the specific plastic polymer types in Malawi, existing studies have reported high levels of single use plastic waste such as carrier bags (Mayoma et al. 2019; Turpie et al. 2019). This aligns with our findings, as the LDPE identified in this study was predominantly made up by single-use plastic bags, reflecting the fact that 80% of the plastics produced in Malawi are single use (Turpie et al. 2019), and predominantly consisting of thin LDPE.,. Other studies in some parts of Asia and Europe have also identified LDPE as high contributors of plastic waste (24%), followed by PET (20%) (Khatib et al. 2023; Lyshtva et al. 2025), which contradict our findings. These differences can be due to the difference in socio economic status of users and recycling and reuse activities within these settings.

### Impacts of rainfall on plastic quantities

**Fig. 10:**
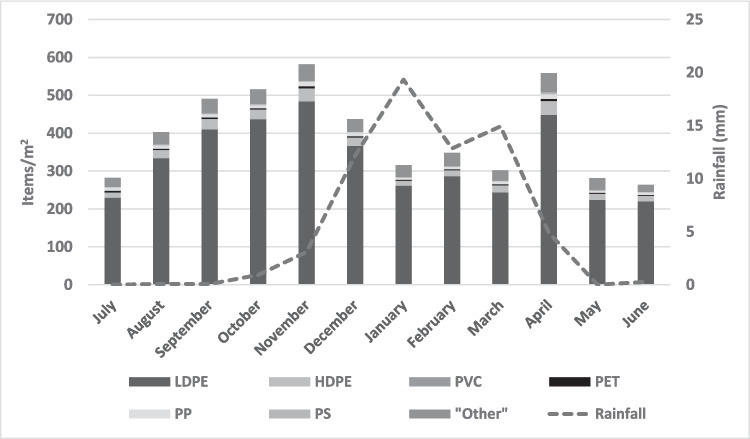
Summary of cumulative plastic counts (individual items) and rainfall over a 12-month period from 14 waste piles in Ndirande

Overall, plastic items were found within the piles throughout the year, with an observed accumulative pattern in the dry season (July-November), as these waste piles accumulate in the absence of waste collection services. Then a decrease in plastic items/(m^2^) is observed during the months experiencing high rainfall (December 2022 to March 2023), with the highest reduction occurring during periods of peak rainfall (January-March) (Fig. 10). This can be attributed to the buoyant and lightweight properties of plastic waste which enables it to be easily carried by rainfall runoff in these waste piles, especially due to the waste pile position (slanted) along rivers enabling plastic runoff.

Other studies have linked normal rainfall and flooding to the creation of large waste quantities within households and aiding in the movement of microplastics (Gichamo & Gökçekuş, 2019; Severe et al. 2025; Xia et al. 2020) highlighting the significant role of rainfall in plastic transport. Similarly, this study links rainfall with plastic dispersion from waste piles, evident in the reduced plastic counts during the rainy season and even worse during extensive flooding. This was exemplified by flooding caused by the tropical cyclone “Freddy” which affected Southern Africa including Malawi in March 2023, which also resulted in some waste piles being completely washed away. Such effects of rainfall on waste, especially the lightweight plastics, calls for enhanced containment strategies of waste within community environments to limit the effects of pollution and contamination in communities (direct and downstream), and surface water sources.

### Plastics as a proportion of all waste

Finally, we wanted to explore the patterns of plastic waste as a proportion of all waste, and how these might differ by month and waste pile location. Below we present results separately for surface and compacted samples of plastics.

#### Surface plastics

**Fig. 11:**
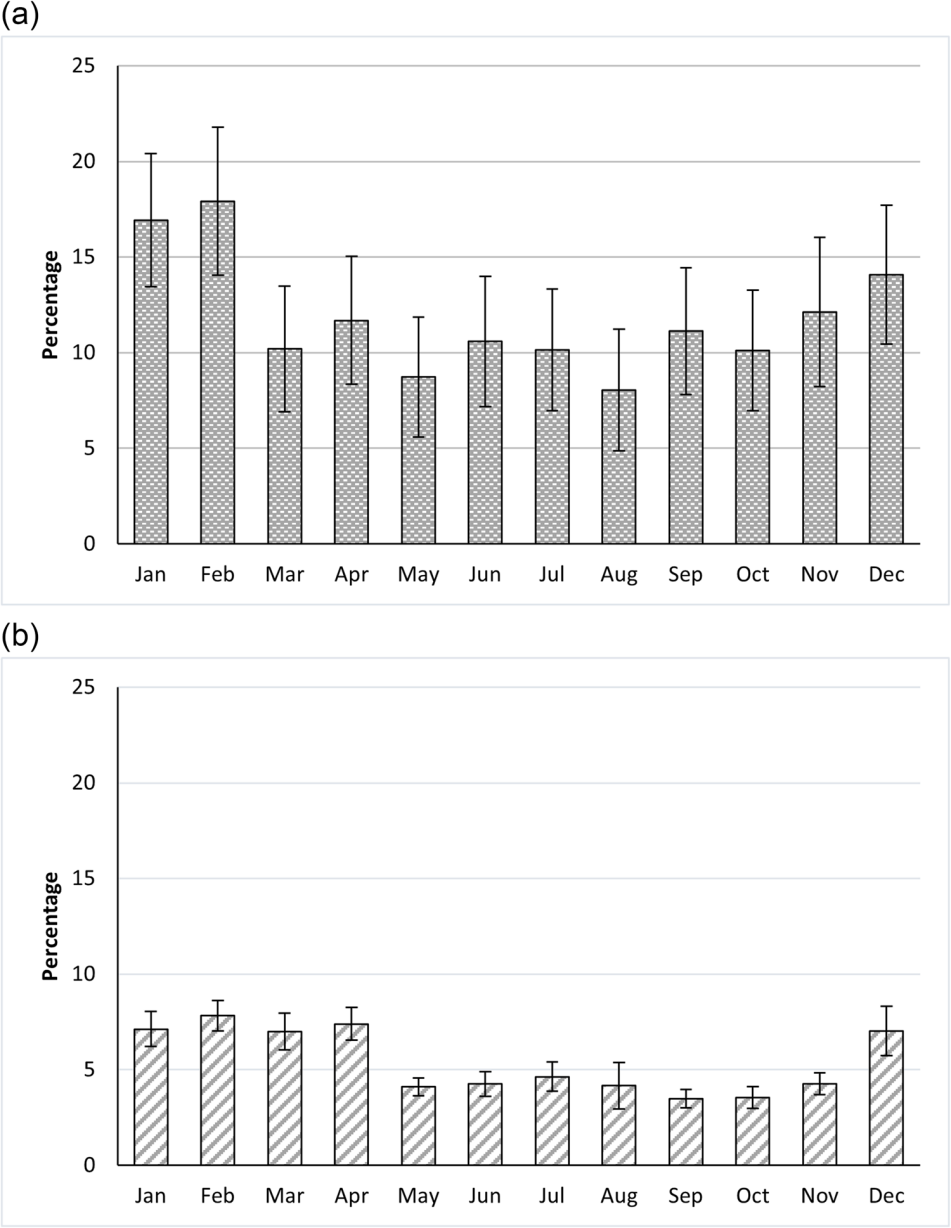
Plastics as a proportion (mean) of total weight (‘percentage’) of (**a**) surface, and (**b**) compacted samples by month

There was a significant difference in the number of plastics, as a proportion of the total waste, on the surface of waste piles by month (F_(11, 14)_ = 4.092, p = 0.008; Fig. 11a), but this was not significantly different between the waste pile locations (F_(2, 13.7)_ = 3.224, p = 0.071) (Supplementary material S10a). There was no interaction effect between month and waste pile location (F_(22, 10.9)_ = 1.339, p = 0.316), so this interaction term was removed from the models. In terms of the month effect, plastics were more prominent (ca. 7%) during December 2022 to April 2023, but lower (ca. 4%) between May and November. Therefore, although plastics showed an accumulation over the dry season (above in Sect. "Results and discussion","Study limitation"), plastics were actually lower as a proportion of all waste during this period. This is likely due to organic waste being more prevalent during the dry season, associated with the harvesting, presence of volumes of maize husk at this time. Adjusting for mean monthly rainfall attenuated the association between month and plastic proportions, although it remained significant at the 5% significance level (F_(10, 14.54)_ = 2.753, p = 0.039). Adjusting for mean monthly temperature had no attenuation effect on the month effect (F_(10, 14.7)_ = 4.246, p = 0.006) and adjusting for rainfall and temperature together in the model also revealed no additional attenuation compared to the model with only mean monthly rainfall included (F_(9, 13.3)_ = 2.923, p = 0.038). Using total monthly rainfall instead of mean monthly rainfall made minimal difference to using average monthly rainfall (F_(10, 14.3)_ = 2.810, p = 0.037). This suggests that rainfall, in part, explains why we see plastics representing a greater proportion of waste during the dry season, as the rain will wash other materials like organics and soil out of the waste piles. However, the fact that the month effect remains after adjustment for rainfall and temperature, although attenuated, suggests there are other explanations, which need further investigation, as to how plastics represent a higher proportion of the surface of these waste piles during the dry season compared to the rainy season.

#### Compacted plastics

From the compacted areas of the waste piles, there was a significant difference by month (F_(11, 13.9)_ = 7.626, p < 0.001) in the number of plastics as a proportion of all waste types (Fig. 10b), but no difference between waste pile location types (riverline, stream or open-ground) (F_(2, 6.4)_ = 0.253, p = 0.784) (Supplementary material S10b). There was no interaction effect between month and waste pile location (F_(22, 11.2)_ = 1.146, p = 0.421), so this interaction term was removed from the models. In terms of the month effect, In terms of the monthly differences, plastics made up a greater proportion of the total waste in January and February (ca. 17%) but a lower proportion from March to December. (Fig. 10b). Adjusting for mean monthly rainfall attenuated the association between month and plastic proportions (F_(10, 18.9)_ = 2.310, p = 0.056). Adjusting for rainfall and temperature together in the same model attenuated this relationship further(F_(9, 18.8)_ = 1.652, p = 0.171). Using total monthly rainfall instead of mean monthly rainfall produced a similar attenuation effect (F_(9, 19)_ = 1.823, p = 0.129). In both models, rainfall was the main driver for the attenuation of the monthly effect compared to temperature, which had no attenuation impact when included in the model without rainfall data (F_(10, 17.4)_ = 8.239, p < 0.001).

## Study limitation

This study has some limitations. The sampling was conducted at a limited number of waste piles, which may not fully represent variability across all informal dumpsites in Malawi. The study relied on grab samples (surface and compacted layers), and therefore may not capture the full depth heterogeneity of waste piles. The study did not link the findings to other parameters of waste such as chemical and physical factors which may also affect the waste pile dynamics. Lastly, the study observed waste pile growth through the length and width measurement and without the height, as this was hard to measure due to the nature of the waste piles. Despite these limitations, the study provides one of the few longitudinal datasets on waste composition in informal waste sites in SSA and Malawi and offers important evidence for improving waste management planning. Future research should consider large scale monitoring of waste composition across different settings, and detailed biochemical analysis of waste fractions to understand environmental risks. Additional work assessing household waste-generation behavior, spatial patterns of waste accumulation, and the performance of targeted interventions (e.g., composting or plastic recovery schemes) would further strengthen the evidence base for waste-management planning in Malawi.

## Conclusion

Our study has identified key areas of waste disposal in a typical informal settlement in sub-Saharan Africa. In this longitudinal study, we mapped 56 informal communal waste piles located alongside rivers and streams, and in open spaces, all of which were located within 10 m of households. The 14 waste piles that were characterised in temporal detail were mainly composed of fine particulates, organic materials, plastics, textiles, and disposable sanitary products. Plastics were the third most common waste item by weight (8.1%), and the second by volume (23.6%), with LDPE (83%) being the most prevalent plastic polymer type. Proportions of waste recovered were affected by month, waste type, and changes in seasonality, and there were variations in the abundance of waste categories between the surface and compacted waste sections of the pile. Our findings underscore the significant role of rainfall in the movement and dispersion of plastics, leading to increased accumulation in natural ecosystems and associated human health risks as well as the significant role of waste material type and waste pile section, which informs the waste pile dynamics.

These findings highlight the urgent need to strengthen strategic waste management initiatives, including the introduction of controlled disposal sites within informal urban communities, waste segregation at source, organic composting, and the inclusion of plastics, particularly LDPE in global and national circular economy models, alongside context-specific regulatory frameworks for single-use plastics. Malawi can leverage the existing thin plastic ban by strict enforcement of the ban to ensure that only recommended plastics are produced in the country, which can then be repurposed or recycled. However, there is also a need for upscaling the existing biodegradable alternatives such as paper bags, by making them affordable and easily accessible, as well as introducing new competitive sustainable alternatives that will meet the needs of people for easy adoption in communities. Seasonal variations and differences between surface and compacted waste layers should also be considered when planning collection schedules and waste recovery interventions. Furthermore, high-risk areas, such as rivers and streams, require targeted clean-up and prevention measures to protect both environmental and public health. In addition, engaging communities in awareness programs about the environmental and human health impacts of solid waste, promoting material-specific recycling, and integrating technologies such as drones for mapping informal disposal sites will enhance the effectiveness and sustainability of these interventions. Overall, addressing solid waste management and plastic pollution is essential not only for protecting vulnerable communities and ecosystems but also for safeguarding biodiversity, ecosystem health, and planetary boundaries.

## Supplementary Information

Below is the link to the electronic supplementary material.

Supplementary file1 (DOCX 164 kb)

Supplementary file2 (DOCX 91 kb)

Supplementary file3 (DOCX 23 kb)

Supplementary file4 (DOCX 19 kb)

Supplementary file5 (DOCX 15 kb)

Supplementary file6 (DOCX 20 kb)

Supplementary file7 (DOCX 19 kb)

Supplementary file8 (DOCX 112 kb)

Supplementary file9 (DOCX 147 kb)

Supplementary file10 (DOCX 95 kb)

## Data Availability

Data can be made available upon reasonable request.
