# Supplementary material for: Mapping and quantifying the spatial and temporal composition of waste piles in informal settlements of urban Malawi
Source: Environ Sci Pollut Res Int. 2026 Mar 7;33(10):4484–502. doi: 10.1007/s11356-026-37534-0 (PMC13053368; doi:10.1007/s11356-026-37534-0)
Supplement: Supplementary file 2 — Supplementary file2 (DOCX 91 kb) [file 11356_2026_37534_MOESM2_ESM.docx]

**Supplementary Material**

**Mapping and quantifying the spatial and temporal composition of waste piles in informal settlements of urban Malawi.**

(a)

**
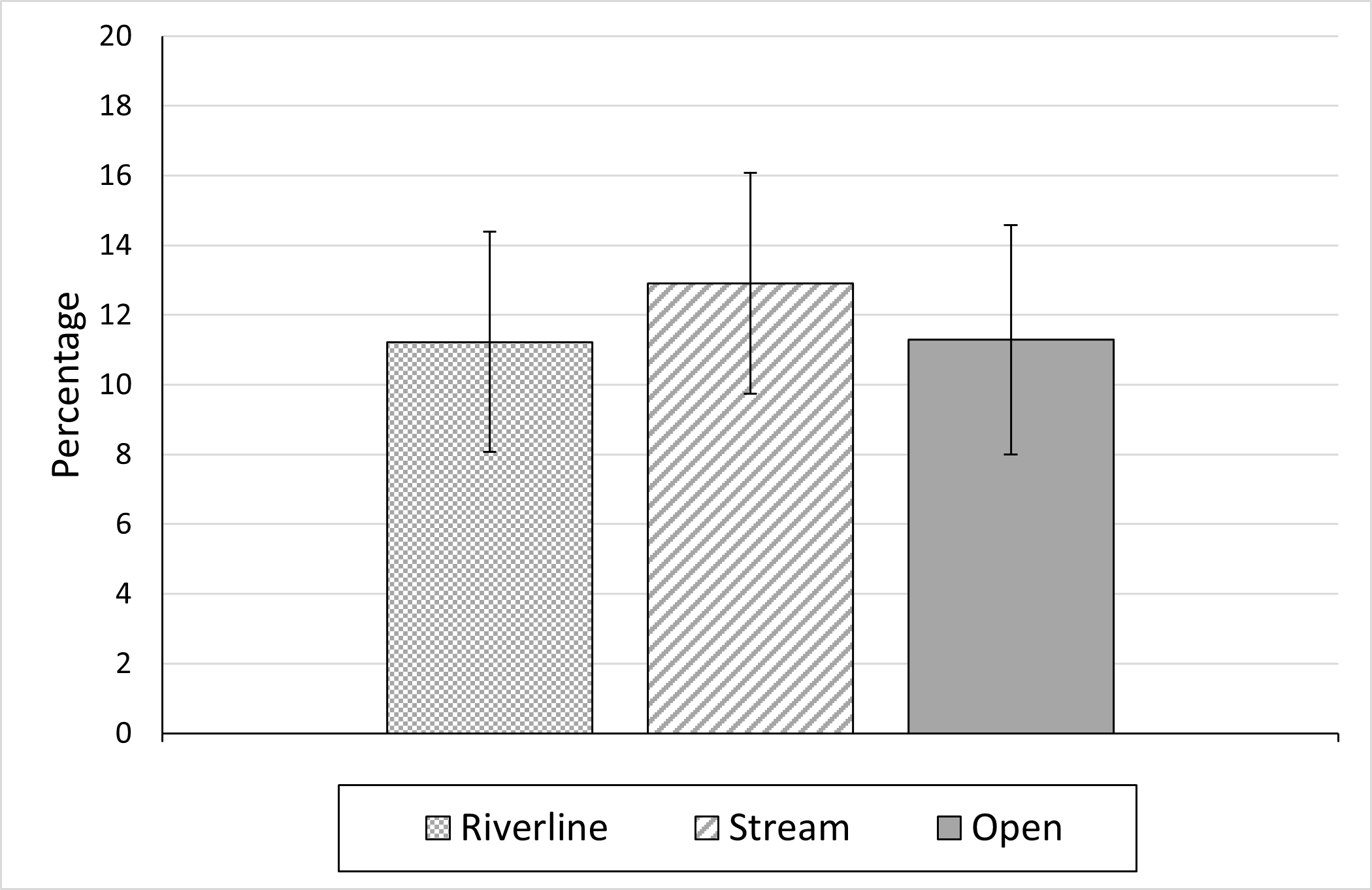
**

(b)

**
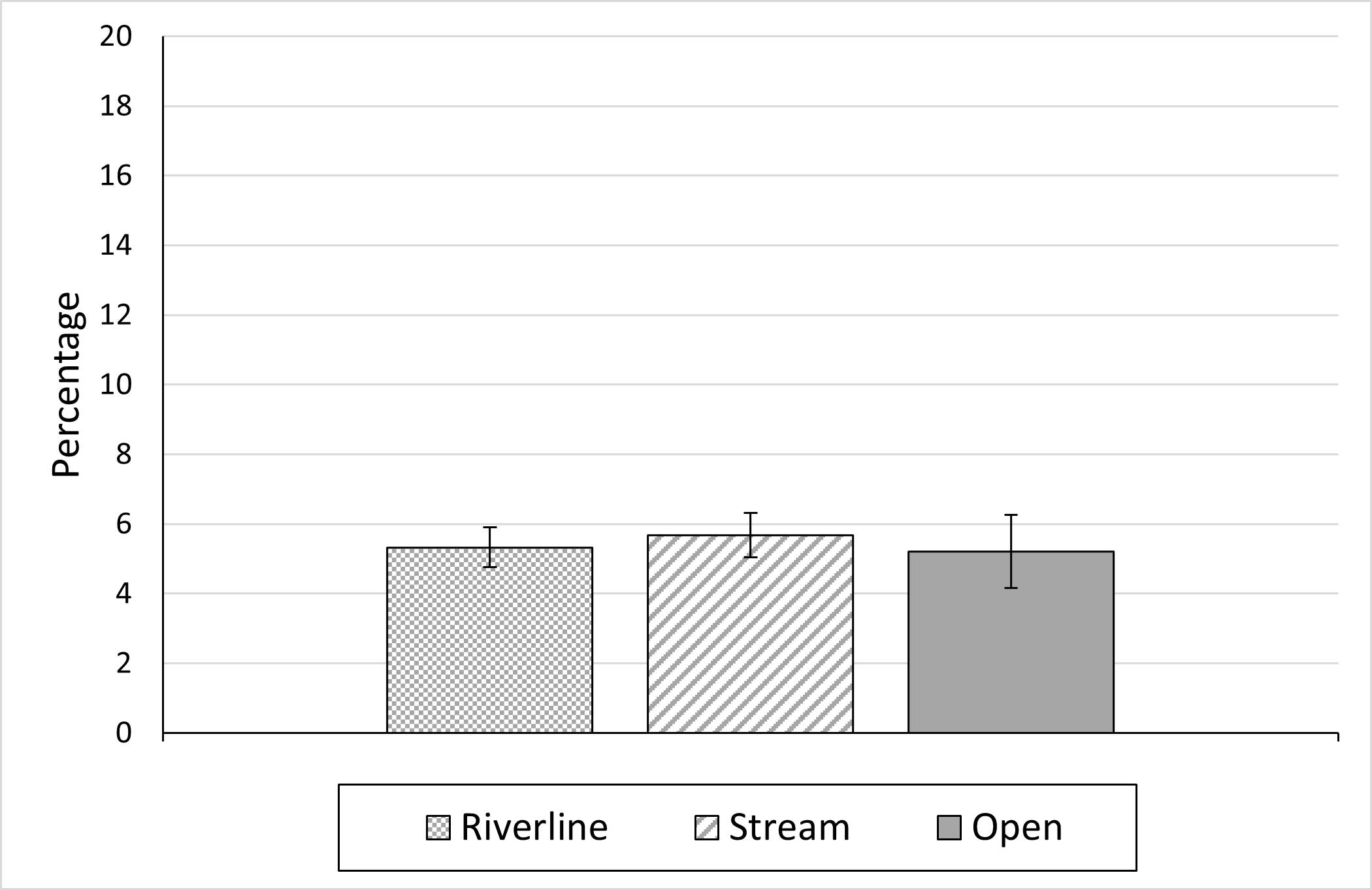
**

**S10:** Plastics as a proportion of total weight of (a) compacted, and (b) surface samples by waste pile type (river line, stream or open).
