# Supplementary material for: Mapping and quantifying the spatial and temporal composition of waste piles in informal settlements of urban Malawi
Source: Environ Sci Pollut Res Int. 2026 Mar 7;33(10):4484–502. doi: 10.1007/s11356-026-37534-0 (PMC13053368; doi:10.1007/s11356-026-37534-0)
Supplement: Supplementary file 5 — Supplementary file5 (DOCX 15 kb) [file 11356_2026_37534_MOESM5_ESM.docx]

**Supplementary material**

**Mapping and quantifying the spatial and temporal composition of waste piles in informal settlements of urban Malawi**

**S4:** Occupied waste volume across waste categories

| **Category** | **Mean**  **(m^3^ (L))** | **SD**  **(m^3^ (L))** | **Min**  **(m^3^ (L))** | **Max**  **(m^3^ (L))** |
| --- | --- | --- | --- | --- |
| Plastics | 0.63 (631.92) | 0.16 (159.65) | 0.45 (447.00) | 0.87 (865.50) |
| Organics | 0.99 (994.13) | 0.29 (291.99) | 0.44 (442.00) | 1.42 (1415.00) |
| Sanitary | 0.06 (67.33) | 0.03 (29.65) | 0.03 (32.50) | 0.13 (131.00) |
| Metals | 0.01 (11.40) | 0.00 (2.79) | 0.01 (7.00) | 0.01 (14.50) |
| Glass | 0.01 (12.04) | 0.00 (2.32) | 0.01 (8.50) | 0.02 (16.50) |
| Paper/Cardboard | 0.13 (125.83) | 0.06 (55.98) | 0.04 (36.50) | 0.22 (215.00) |
| Textile | 0.18 (187.63) | 0.05 (48.96) | 0.14 (137.50) | 0.28 (277.00) |
| Foil | 0.03 (31.33) | 0.02 (18.86) | 0.01 (14.00) | 0.08 (84.00) |
| Soil and fines | 0.51 (512.89) | 0.20 (199.44) | 0.24 (235.00) | 0.72 (720.00) |
| Other | 0.08 (85.92) | 0.02 (23.31) | 0.06 (61.00) | 0.12 (124.50) |
