# Supplementary material for: Mapping and quantifying the spatial and temporal composition of waste piles in informal settlements of urban Malawi
Source: Environ Sci Pollut Res Int. 2026 Mar 7;33(10):4484–502. doi: 10.1007/s11356-026-37534-0 (PMC13053368; doi:10.1007/s11356-026-37534-0)
Supplement: Supplementary file 6 — Supplementary file6 (DOCX 20 kb) [file 11356_2026_37534_MOESM6_ESM.docx]

**Supplementary material**

**Mapping and quantifying the spatial and temporal composition of waste piles in informal settlements of urban Malawi**

**S5:** Plastic quantities (items/m^2^) across all waste piles
