# Supplementary material for: Mapping and quantifying the spatial and temporal composition of waste piles in informal settlements of urban Malawi
Source: Environ Sci Pollut Res Int. 2026 Mar 7;33(10):4484–502. doi: 10.1007/s11356-026-37534-0 (PMC13053368; doi:10.1007/s11356-026-37534-0)
Supplement: Supplementary file 8 — Supplementary file8 (DOCX 112 kb) [file 11356_2026_37534_MOESM8_ESM.docx]

**Supplementary Material**

**Mapping and quantifying the spatial and temporal composition of waste piles in informal settlements of urban Malawi.**

**
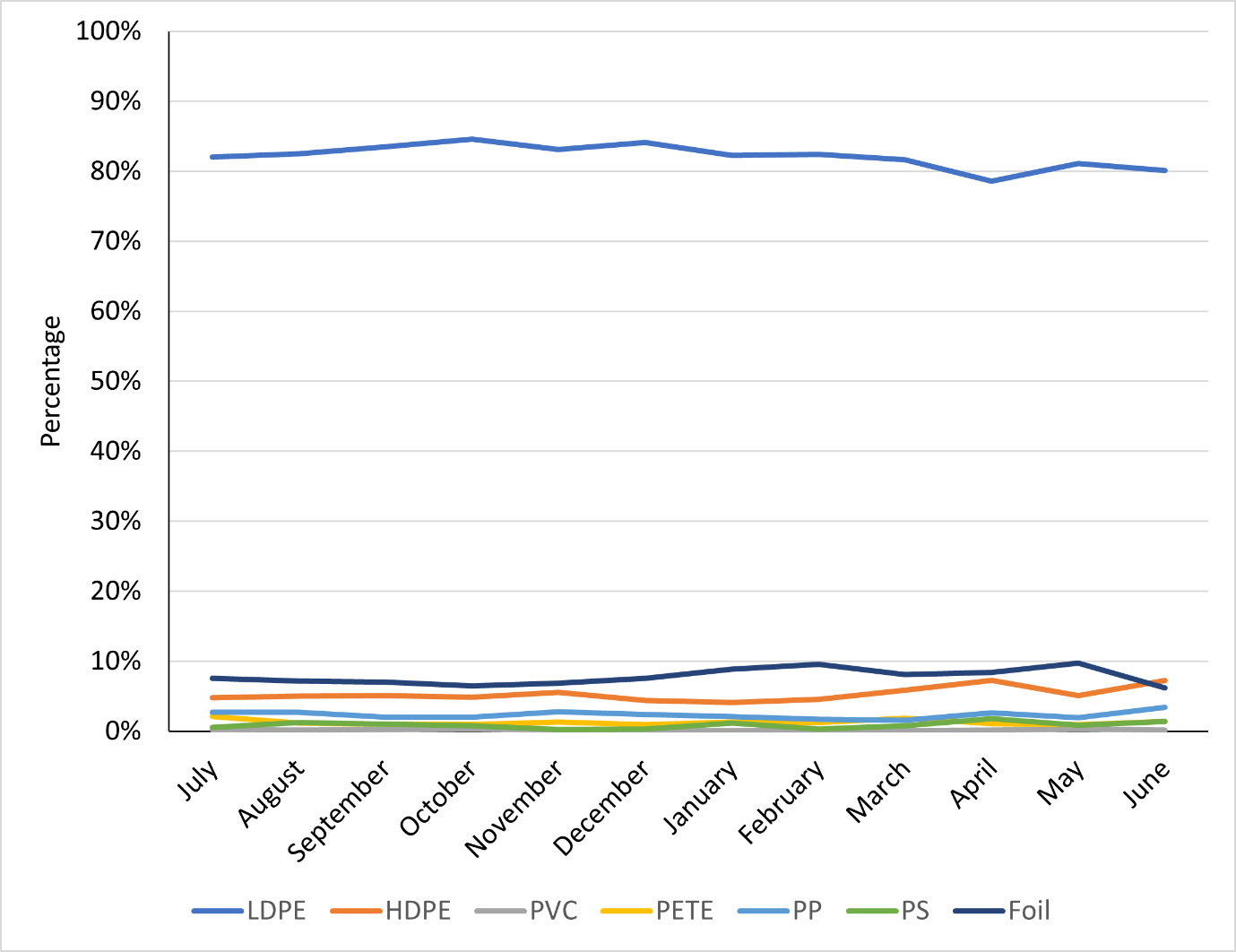
**

**S6**     Proportion of plastic types (percentage by item/m^2^) in combined surface samples by month
